# Prevalence of presbyopia among social safety net beneficiaries with the cognitive, numeracy and dexterity skills required for smartphone use: a cross-sectional analysis of THRIFT RCT screening data from Kurigram, Bangladesh

**DOI:** 10.1136/bmjopen-2025-108327

**Published:** 2026-04-07

**Authors:** Ishrat Binte Aftab, Tisha Chakma, Sonia Pant, Lovemore Nyasha Sigwadhi, Sharmin Akter Shitol, H M Masudur Rahman, Jahangir Alam, Enam Haque, Harithaa P Chadalavada, Fahmeeda Murtaza, Ving Fai Chan, Julie-Anne Little, Rohit C Khanna, Graeme MacKenzie, Ella Gudwin, Lynne Lohfeld, Mike Clarke, Abu Shonchoy, Nathan Congdon, Atonu Rabbani

**Affiliations:** 1 BRAC University James P Grant School of Public Health, Dhaka, Bangladesh; 2 VisionSpring, New York, New York, USA; 3 Division of Epidemiology and Biostatistics, Department of Global Health, Faculty of Medicine and Health Sciences, University of Stellenbosch, Cape Town, Western Cape, South Africa; 4 Clinical Trials Unit, LV Prasad Eye Institute, Hyderabad, India; 5 Centre for Public Health, School of Medicine, Dentistry and Biomedical Sciences, Queen’s University Belfast, Belfast, UK; 6 MOMODa Foundation, Dhaka, Bangladesh; 7 Department of Ophthalmology and Vision Sciences, University of Toronto, Toronto, Ontario, Canada; 8 Centre for Optometry and Vision Science, School of Biomedical Sciences, Ulster University, Coleraine, UK; 9 Allen Foster Community Eye Health Research Centre, Gullapalli Pratibha Rao International Centre for Advancement of Rural Eye care, LV Prasad Eye Institute, Hyderabad, India; 10 Brien Holden Eye Research Centre, LV Prasad Eye Institute, Hyderabad, India; 11 School of Optometry and Vision Science, University of New South Wales, Sydney, New South Wales, Australia; 12 School of Medicine and Dentistry, University of Rochester, Rochester, New York, USA; 13 Riemann Limited, London, UK; 14 Florida International University, Miami, Florida, USA; 15 Zhongshan Ophthalmic Centre, Sun Yat-Sen University, Guangzhou, Guangdong, China; 16 ORBIS International, New York, New York, USA; 17 Department of Economics, Dhaka University, Dhaka, Bangladesh

**Keywords:** OPHTHALMOLOGY, Aged, Prevalence

## Abstract

**Abstract:**

**Objectives:**

To determine the prevalence of presbyopia and associated risk factors among Bangladeshi recipients of elderly social safety net payments who were not currently using mobile financial services (MFS) and demonstrated numeracy, dexterity and cognitive prerequisites for smartphone use during eligibility screening for the Transforming Households with Refraction and Innovative Financial Technology (THRIFT) trial. Accessing these payments requires use of online banking, as with a smartphone.

**Design:**

Cross-sectional analysis of trial eligibility screening data.

**Setting:**

Community-based screening conducted in two rural subdistricts in Kurigram District, Bangladesh.

**Participants:**

Among 13 944 Old Age Allowance and Widows’ Allowance (WA) beneficiaries screened, 953 met trial eligibility criteria, including passing a smartphone readiness assessment and completing near vision examinations.

**Primary and secondary outcome measures:**

Presbyopia, defined as binocular presenting near visual acuity of N6.3 or worse, correctable to at least N5 with near vision glasses and with distance vision of ≥6/12 in both eyes.

**Results:**

Among 953 participants (mean age 61.4±7.2 years, 62.6% women), presbyopia prevalence was 62.6% (95% CI 59.5 to 65.7). Presbyopia was significantly positively associated with female gender (adjusted prevalence ratio (APR)=1.19, 95% CI 1.02 to 1.41) and receiving WA (APR=1.20, 95% CI 1.04 to 1.38) in multivariable analyses.

**Conclusions:**

This study highlights a substantial burden of uncorrected presbyopia among a prescreened, randomised control trial-eligible subgroup of social safety net beneficiaries in rural Bangladesh, who were not currently using MFS but demonstrated cognitive and functional capacity to use mobile phones, potentially hampering their ability to carry out online banking. Delivery of reading glasses may improve digital financial access and facilitate broader financial inclusion, a hypothesis currently being tested in the parent THRIFT trial.

**Trial registration number:**

NCT05510687.

**STRENGTHS AND LIMITATIONS OF THIS STUDY**

- The study used a standardised protocol to assess near vision and functional prerequisites for smartphone use, including cognition, numeracy and dexterity.
- Thus, the presence of presbyopia, a potential barrier to smartphone use, was only assessed among those otherwise capable of using such phones.
- Participants were drawn from a highly relevant group, beneficiaries of government social safety net programmes requiring access through mobile banking.
- The study sample reflects an economically important and vulnerable group receiving government benefits and is not meant to be population-representative.

## Introduction

 Presbyopia, an age-related condition characterised by the progressive loss of near focusing ability, affects nearly all older adults, beginning at around age 35, the peak of the working years.^1^ By 2020, uncorrected presbyopia caused near vision impairment in 510 million people globally, primarily in South, East and Southeast Asia.^2^ Among adults aged 50 and older, an estimated 419 million people around the globe had near vision impairment from uncorrected presbyopia in 2020.^3^ Despite the potential for simple correction with near vision glasses, effective refractive error coverage remains low at just 20.5% among individuals aged 50 and older.^4^

Mobile financial services (MFSs) are improving access to banking services, particularly in low- and middle-income countries (LMICs).^5^ In Bangladesh, the government has integrated MFS into its social safety net programmes for the elderly, including the Old Age Allowance (OAA) and Widows’ Allowance (WA), which provide monthly cash transfers to vulnerable populations. Since 2021, payments have been made exclusively through MFS platforms like bKash and Nagad, potentially improving convenience and safety for recipients.^6^

However, previous research has identified a significant burden of uncorrected presbyopia in Bangladesh.^7^ Evidence from several countries suggests that presbyopia can hinder the use of mobile phones.^8^,^13^ Older adult beneficiaries of OAA and WA may struggle to use the mobile phone due to presbyopia. Thus, understanding the prevalence of presbyopia among this population and addressing the burden is crucial for maximising the financial impact of social safety net programmes targeting the vulnerable elderly.

Transforming Households with Refraction and Innovative Financial Technology (THRIFT)^14^ is a randomised control trial (RCT) designed to evaluate the impact of providing near-vision glasses and basic digital financial training on presbyopic OAA and WA beneficiaries’ use of mobile banking in Bangladesh. Although extensive literature is available on the global burden of uncorrected presbyopia,^2^ there is a need for specific data on the burden among particular vulnerable populations with an inherent strong need for near vision, such as older persons with a requirement to use smartphones for online banking. The current study is designed to fill this evidence gap by focusing on OAA and WA recipients who are required to access payments through online banking. The objective of the current paper is to determine the prevalence of presbyopia and associated risk factors among OAA and WA beneficiaries eligible for THRIFT,^14^ specifically those who are poor, not currently using MFS and possess the dexterity, numeracy and cognitive ability to use smartphones.

## Methods

### Study design and setting

This study draws data from the THRIFT RCT^14^ screening and recruitment phases in Kurigram Sadar and Nageshwari. These two subdistricts are chosen as OAA and WA allowances are distributed via study partner bKash and vision programmes by BRAC and VisionSpring are active there.

### Study procedures

**Figure 1:**
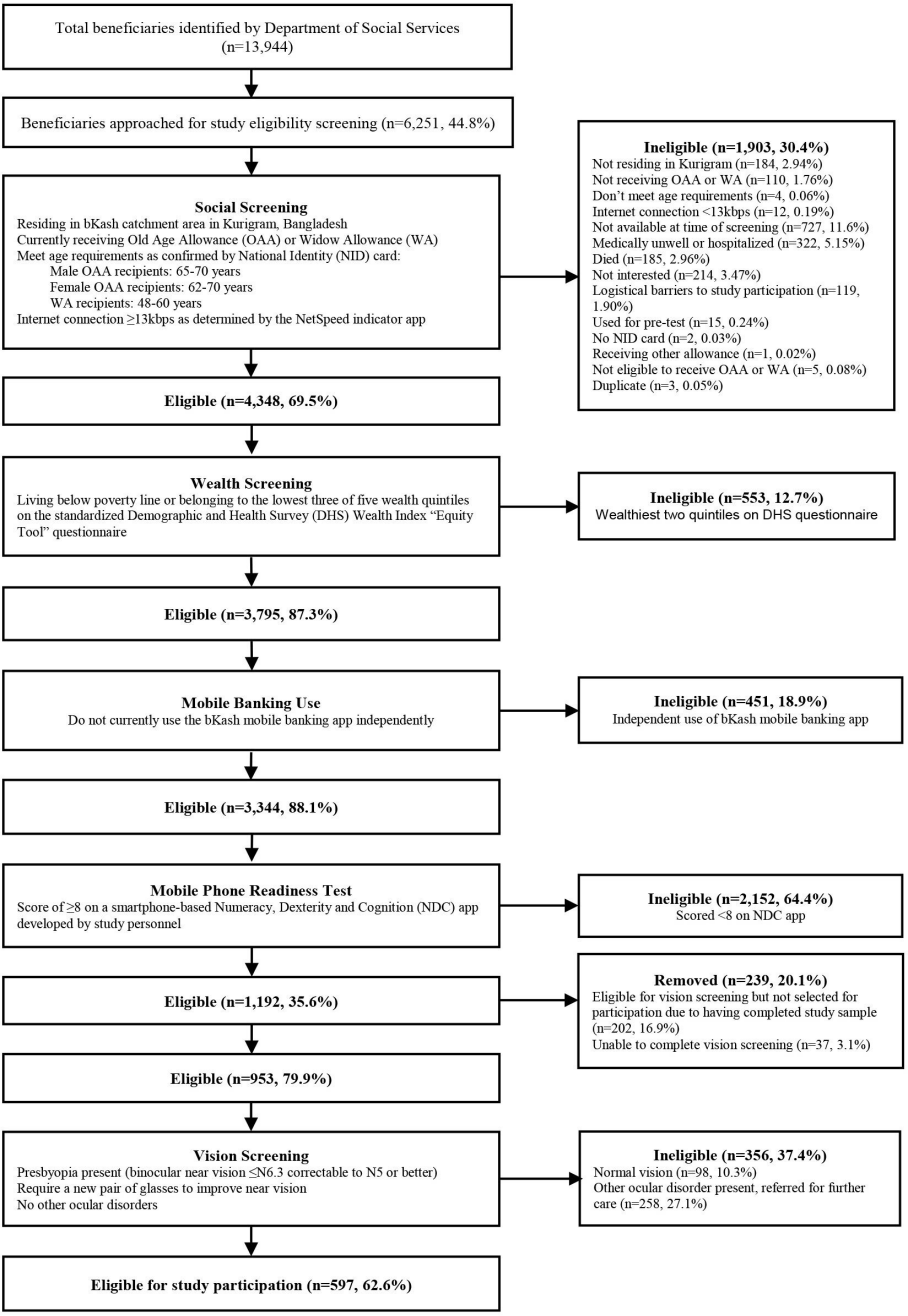
Progress of potential participants through the study.

A structured, multistep screening process was implemented to identify eligible participants for the THRIFT trial (figure 1). Beneficiaries identified from the DSS database were visited door-to-door to assess demographics, socio-economic status, numeracy, manual dexterity and cognitive functioning required to perform basic financial functions on a mobile device. Respondents meeting initial criteria underwent eye examinations for presbyopia, with eligible individuals (meeting all inclusion criteria below) invited to consent for enrolment in THRIFT.^14^ For participants who were unable to read or write, trained enumerators read the full consent form aloud in Bengali, the local language. Participants were encouraged to ask questions, and enumerators confirmed participants understood the content of the consent form before proceeding. Those agreeing to participate provided thumbprint consent in place of a signature.

Race/ethnicity data were not collected as effectively the entire cohort belongs to a single national ethnic group.

#### Social screening

From an initial pool of beneficiaries identified by the DSS, individuals from bKash catchment areas in Kurigram, Bangladesh were selected. Age criteria were 65–70 and 62–70 years for male and female OAA recipients, respectively. For WA recipients, participants were chosen between 48–60 years of age. Additional eligibility requirement assessed at this stage included internet connection speed ≥13 kbps, verified using the NetSpeed indicator application.

#### Wealth screening

Economic eligibility was assessed using an Equity Tool, based on the Demographic and Health Survey Wealth Index.^15 16^ Participants from the lowest three of five wealth quintiles or those living below the poverty line were eligible, while those in the top two quintiles were excluded.

#### Mobile banking use

Participants independently using bKash mobile banking software were excluded.

### Mobile phone readiness test

A smartphone-based numeracy, dexterity and cognition test evaluated participants’ ability to identify numbers when spoken to them in the local language and correctly input them into a touchpad interface, designed to resemble the bKash application. A minimum score of 8 out of 10 digits (0–9, presented in random order) was required for eligibility.

#### Vision screening

Door-to-door visits were conducted by trained community health workers provided lists of persons provisionally eligible based on prior screenings. An external examination of both eyes was conducted to identify conditions, including redness, watery discharge, obvious lens opacity, evidence of ocular injury or pterygium requiring referral. Distance and near visual acuity were measured using tumbling E reduced logarithm of the minimum angle of resolution charts at 3 m and 40 cm, respectively. Presbyopia was defined as binocular presenting near visual acuity of N6.3 or worse, correctable to at least N5 with near vision glasses and with distance vision of ≥6/12 in both eyes.

### Statistical analyses

Categorical variables were analysed using frequencies and percentages. The Shapiro-Wilk test was used for normality assessment. χ² and Fisher’s exact test were used to assess the association between presbyopia and categorical variables. Linear by linear trend tests were conducted for ordered variables to assess trend across age categories, educational level and national quintiles, while χ² tests were used for non-ordered categorical variables to assess trend across the categories.

Due to the high prevalence of presbyopia, around 63%, logistic regression overestimated effect measures with large standard errors and wide CIs. Log-binomial regression had convergence problems, and thus robust Poisson regression was used to assess the significance of associations between presbyopia and potential predictors. Factors with p<0.15 in unadjusted univariable regression were included in a multivariable model. However, mobile type was considered an a priori variable based on this study importance and was included in the multivariable analysis regardless of its p value (0.271). Adjusted prevalence ratios (APRs) with 95% CIs were used to measure association. Factors with p<0.05 were considered significant. All statistical analyses were performed using Stata (V.18, Stata Corp, College Station, Texas, USA).

### Patient and public involvement

Patients and the public were involved in the formative qualitative phase of the study, including workshops to develop the theory of change and inform the study design.

## Results

From an initial pool of 13 944 beneficiaries identified by the DSS, 6251 (44.8%) met residency criteria and were approached for eligibility screening (figure 1). Following social screening, 4348 (69.5%) persons remained provisionally eligible. Of these, 3795 people (87.3%) belong to the lowest three wealth quintiles and meet wealth eligibility criteria.

Additionally, 451 (18.9%) participants were disqualified for prior use of bKash mobile banking software independently, while 2152 (64.4%) were eliminated after failing the mobile phone readiness test, leaving 1192 (35.6%) eligible for vision screening. However, 202 (16.9%) participants were not selected for testing, due to exceeding sample size requirements of the parent trial. Additionally, 37 (3.1%) were removed due to screening failure and wrong vision screening (figure 1).

Among 953 participants included in the current study of near vision assessment, 597 (62.6%, 95% CI 59.5% to 65.7%) were presbyopic. Of all participants, 25.7% were aged 45–54 years, 21.2% were aged 55–64 years and 53.1% were aged 65 years or older (table 1). A majority of participants were women (62.6%). A total of 79.9% had never attended school, and only 4.1% had completed secondary or higher school. Three-quarters of the participants were employed, with 20.9% being retired. Nearly all (97.3%) participants had access to a mobile phone; however, only 5.6% had smartphones, and 7.6% had internet access. 23% of participants were using eyeglasses, and more than half were in the lowest wealth quintile among those approached for eligibility screening in this current study. A total of 515 (54.0%) were receiving the OAA, while the rest received WA.

**Table 1:** Descriptive statistics of the study participants

Characteristic	Total (n=953)	Presbyopic (597)	Non-presbyopic (n=356)	P value
Age (years)				<0.001
45–54	245 (25.7)	183 (30.7)	62 (17.4)	
55–64	202 (21.2)	138 (23.1)	64 (18.0)	
65+	506 (53.1)	276 (46.2)	230 (64.6)	
Sex				<0.001
Male	356 (37.4)	183 (30.7)	173 (48.6)	
Female	597 (62.6)	414 (69.3)	183 (51.4)	
Highest level of education				0.11
Never attended school	761 (79.9)	487 (81.6)	274 (77.0)	
Completed primary school	153 (16.1)	90 (15.1)	63 (17.7)	
Completed secondary school	34 (3.6)	19 (3.2)	15 (4.2)	
Completed higher secondary school	5 (0.5)	1 (0.2)	4 (1.1)	
Literacy level				0.022
Unable to read or write	684 (71.8)	447 (74.9)	237 (66.6)	
Can read	30 (3.1)	16 (2.7)	14 (3.9)	
Can read and write	239 (25.1)	134 (22.4)	105 (29.5)	
Occupation				0.081
Unemployed	34 (3.6)	25 (4.2)	9 (2.5)	
Employed	720 (75.6)	459 (76.9)	261 (73.3)	
Retired	199 (20.9)	113 (18.9)	86 (24.2)	
Currently have access to a mobile phone				0.91
Yes	927 (97.3)	581 (97.3)	346 (97.2)	
No	26 (2.7)	16 (2.7)	10 (2.8)	
Who owns the phone?				0.25
My own	602 (64.9)	384 (66.1)	218 (63.0)	
Family member	300 (32.4)	185 (31.8)	115 (33.2)	
Neighbour	25 (2.7)	12 (2.1)	13 (3.8)	
Mobile phone type				0.31
Smartphone	52 (5.6)	36 (6.2)	16 (4.6)	
Other	875 (94.4)	545 (93.8)	330 (95.4)	
Internet connection on the phone				0.63
Yes	70 (7.6)	42 (7.2)	28 (8.1)	
No	857 (92.4)	539 (92.8)	318 (91.9)	
Eyeglasses use				0.36
Yes	219 (23.0)	143 (24.0)	76 (21.3)	
No	734 (77.0)	454 (76.0)	280 (78.7)	
Wealth quintile				0.13
Quintile 1	516 (54.1)	313 (52.4)	203 (57.0)	
Quintile 2	269 (28.2)	182 (30.5)	87 (24.4)	
Quintile 3	168 (17.6)	102 (17.1)	66 (18.5)	
Beneficiary type				<0.001
OAA	515 (54.0)	280 (46.9)	235 (66.0)	
WA	438 (46.0)	317 (53.1)	121 (34.0)	

Notes: variables were expressed as frequencies with percentages in parentheses. Data for all variables were available for all participants.

OAA, Old Age Allowance; WA, Widows’ Allowance.

Persons with presbyopia were significantly older, more likely to be female and illiterate than those without (table 1). The highest prevalence of presbyopia was observed among individuals aged 45–54 years (74.7%), with a significant decrease in prevalence with increasing age (test for trend, p<0.001, table 2). Presbyopia was more prevalent in women (p<0.001), those with lower education (p=0.056) and WA beneficiaries (p<0.001) (table 2). In multivariable regression models, female gender and WA status remained significant predictors of higher presbyopia risk (table 3). Women were 19% more likely to have presbyopia than men (APR=1.19, 95% CI 1.02 to 1.41, p=0.031, table 3). Beneficiaries of WA were more presbyopic than those receiving OAA, APR=1.20 (95% CI 1.04 to 1.38, p=0.010, table 3).

**Table 2:** Prevalence of presbyopia by demographic and socioeconomic characteristics of study participants

Characteristic	Number	Prevalence (95% CI)	Trend test for association
Age (years)			<0.001
45–54	183	74.7 (68.8 to 80.0)	
55–64	138	68.3 (61.4 to 74.7)	
65+	276	54.5 (50.1 to 58.9)	
Sex*			<0.001
Male	183	51.4 (46.1 to 56.7)	
Female	414	69.3 (65.5 to 73.0)	
Highest level of education			<0.056
Never been to school	487	64.0 (60.5 to 67.4)	
Completed primary school (grade five pass)	90	58.8 (50.6 to 66.7)	
Completed secondary or higher school certificate	20	51.3 (34.8 to 67.6)	
Wealth quintile			0.540
Quintile 1	313	60.7 (56.3 to 64.9)	
Quintile 2	182	67.7 (61.7 to 73.2)	
Quintile 3	102	60.7 (52.9 to 68.1)	
Beneficiary type*			<0.001
OAA	280	54.4 (50.0 to 58.7)	
WA	317	72.4 (67.9 to 76.5)	
Total	597	62.6 (59.5 to 65.7)	

NB: non-parametric tests for trend for ordered categorical variables.

* χ² test was used to assess the group comparison for non-ordered categorical variables.

OAA, Old Age Allowance; WA, Widows’ Allowance.

**Table 3:** Factors associated with presbyopia, univariate and multivariable analysis

Variable	Univariate		Multivariable	
	PR (95 % CI)	P value	APR (95% CI)	P value
Age (years)				
45–54	Ref			
55–64	0.91 (0.81 to 1.03)	0.141		
65+	0.73 (0.66 to 0.81)	<0.001		
Sex				
Male	Ref			
Female	1.35 (1.20 to 1.51)	<0.001	1.19 (1.02 to 1.41)	0.031
Highest level of education				
Never been to school	Ref			
Completed primary school (grade five pass)	0.92 (0.80 to 1.06)	0.248		
Completed secondary/higher school certificate	0.80 (0.59 to 1.09)	0.162		
Occupation				
Unemployed	Ref			
Employed	0.87 (0.70 to 1.07)	0.181		
Retired	0.77 (0.61 to 0.98)	0.031		
Mobile type				
Feature or button	Ref			
Smartphone	1.11 (0.92 to 1.34)	0.271	1.10 (0.91 to 1.33)	0.314
Eyeglasses use				
No	Ref			
Yes	1.06 (0.94 to 1.18)	0.343		
Wealth quintile				
Quintile 1	1.00 (0.87 to 1.15)	0.990	0.96 (0.83 to 1.11)	0.608
Quintile 2	1.11 (0.96 to 1.29)	0.149	1.11 (0.95 to 1.29)	0.189
Quintile 3	Ref		Ref	
Beneficiary type				
OAA	Ref			
WA	1.33 (1.21 to 1.47)	<0.001	1.20 (1.04 to 1.38)	0.010

Notes: PRs and APRs are presented with their 95% CIs in parentheses.

APRs, adjusted prevalence ratios; OAA, Old Age Allowance; PRs, prevalence ratios; WA, Widows’ Allowance.

## Discussion

This study aims to assess the prevalence of presbyopia among a population-based sample of OAA and WA beneficiaries in the THRIFT trial,^14^ possessing the dexterity, numeracy and cognitive ability required for smartphone use. To our knowledge, no other population-based studies examine the prevalence of presbyopia among older online banking users in lower-middle income countries. Using data from the THRIFT^14^ screening and recruitment phases, our findings indicate a high prevalence of presbyopia, an important potential barrier to smartphone use for MFS, in this cohort who are required to use online banking to access government-provided old age benefits.

In this study, the observed prevalence of presbyopia was 62.6%, consistent with studies in rural Tanzania (61.7%),^17^ rural China (67.3%),^18^ South India (61.8%)^19^ and Nigeria (67.3%).^20^ Another study, following the Rapid Assessment of Refractive Error (RARE) methodology, reported a similar prevalence (62%) in Sirjaganj, northern Bangladesh, where presbyopia was assessed exclusively among individuals aged 35 and older.^7^ However, a study carried out in Durban, South Africa, found a higher prevalence of presbyopia (77%) and in a suburban community in South-West Nigeria, the prevalence was 75%.^21 22^ Conversely, the prevalence in our study was higher than that reported in northwest Nigeria (30.4%) and India (42.9%).^23 24^

The varying prevalence in these studies may reflect different definitions of presbyopia, age distributions of the study populations and assessment methods including the type of near vision chart used, testing conditions and test distances. Some studies included participants aged ≥35 years,^7 22 24^ others used a cut-off of ≥40 years^17^,^1921 23^ and one study used ≥30 years among cosmetologists.^20^ Participants were aged 48–70, aligning with the government’s criteria for OAA and WA beneficiaries. The higher presbyopia rates in South Africa and Nigeria may relate to a greater prevalence of hyperopia (highest global prevalence) in African populations,^25^ though we lack data to confirm this.

A surprising finding in the present study was the decreasing prevalence of presbyopia with age. In contrast, Burke *et al* found the opposite trend, which is also reported in other studies.^17 21 22 24 26^ However, Mashayo *et al*^27^ observed an increasing trend in the prevalence of presbyopia from the ages of 35–74 years, following a decreasing trend among individuals aged 75 and above. The decline in presbyopia per se among older individuals is widely reported and is inherent in the definition, which requires that persons with impaired near vision should have normal distance vision, to distinguish between persons with conditions such as cataract, also prevalent in older populations, which affect both near and distance vision. Thus, as the burden of cataract rises in older cohorts, the prevalence of pure presbyopia declines as a direct consequence.^28^ Among participants aged 65 and older, only 54.1% had presbyopia, while 34.4% were referred for additional care due to other vision issues, and 11.1% had normal vision. However, age was not included in our multivariable model as the beneficiary type (WA vs OAA) was strongly associated with age and was included instead.

Our findings indicate the prevalence of presbyopia is significantly higher among women, even after adjustment for other factors. Several previous studies also found higher prevalence of presbyopia among women,^17 21 23 26^ including a RARE study in Bangladesh.^7^ Pointer suggested, possibly due to physiological and physical differences, women typically need more near vision correction than men of the same age.^29^ However, a meta-analysis suggested women’s increased risk of presbyopia may stem from task-related factors and viewing distances.^30^ Another alternative explanation could be lower access to education, which tends to promote myopia in women and girls. Further research is needed to explore these gender differences.

We found higher presbyopia prevalence among less educated participants, consistent with findings from weaving communities in Andhra Pradesh, India^19^ and Southwest Nigeria,^26^ though education-related patterns vary across studies. Similarly, Laviers *et al*^31^ found out that functional presbyopia was associated with lower literacy levels, particularly in rural regions. These findings may be due to a higher prevalence of myopia among more highly educated persons. However, several previous studies reported a higher prevalence of presbyopia among those with greater educational attainment, as seen in Tanzania,^17 27^ Northwest Nigeria^23^ and India.^24^ The higher prevalence of presbyopia among more educated persons may be related to higher visual demand for near tasks such as reading^23^ and will depend on the specific means of case definition and ascertainment.

We found no significant association between wealth and prevalence of presbyopia. This may be because social safety net beneficiaries are predominantly from the most economically deprived strata of society, lacking the broader social distribution that might highlight such an association. Few previous studies include economic status among the potential risk factors for presbyopia.

We observed a high overall prevalence of presbyopia (62.6%) among social safety net beneficiaries, including WA (72.4%) and OAA beneficiaries (54.4%). This significant burden of uncorrected presbyopia likely hinders mobile use,^18^ including mobile banking, despite beneficiaries in our study having the necessary numeracy, dexterity and cognitive skills. Addressing uncorrected presbyopia could improve access to funds, as all social benefits in Bangladesh are paid via MFSs. Our THRIFT trial^14^ is designed to address the hypothesis that providing near-vision glasses and basic digital financial training improves presbyopic beneficiaries’ use of mobile banking in Bangladesh. Correcting presbyopia has other significant economic and social benefits. For instance, RCTs in India and Bangladesh have shown that providing reading glasses enhances work productivity by 22% and increases income by 33.4%.^32 33^ Uncorrected presbyopia caused an estimated US$54 billion in productivity loss in LMICs alone in 2019.^34^

A key strength of this study is its focus on uncorrected presbyopia in a unique and highly relevant group, potential recipients of government benefits in Bangladesh, delivered exclusively through mobile banking. This cohort of OAA/WA beneficiaries was assessed with standardised protocol^14^ and complete data, systematically excluding other barriers like limited cognition, numeracy and dexterity.

A limitation is that participants represented a prescreened, RCT-eligible cohort of OAA/WA beneficiaries who are poor, not currently using MFSs and cognitively and functionally capable of using a smartphone—a highly selected subgroup, therefore, does not provide population-representative sample. Nonetheless, they constitute a group with a unique need to access MFSs. These results are representative only of this cohort and geographical region of Bangladesh, and our results cannot be applied to other settings with confidence. Moreover, given the limited relevance of systemic comorbidities to the primary study outcome, we have not chosen to include data on comorbidities in our analyses. Future investigations can include comprehensive health assessments to explore potential effect modification.

Despite its limitations, the current study provides strong evidence of the burden of uncorrected presbyopia in a cohort of persons requiring access to mobile banking, with its inherent demands on near vision.^35^ The ongoing THRIFT trial^14^ will determine whether alleviating this burden results in greater financial inclusiveness among this vulnerable group of older safety net beneficiaries in Bangladesh.

## Data Availability

Data are available upon reasonable request.
