# Supplementary material for: Prevalence of presbyopia among social safety net beneficiaries with the cognitive, numeracy and dexterity skills required for smartphone use: a cross-sectional analysis of THRIFT RCT screening data from Kurigram, Bangladesh
Source: BMJ Open. 2026 Apr 7;16(4):e108327. doi: 10.1136/bmjopen-2025-108327 (PMC13064153; doi:10.1136/bmjopen-2025-108327)
Supplement: Supplementary file 1 [file bmjopen-2025-108327.reviewer_comments.pdf]

PEER REVIEW HISTORY

BMJ Open publishes all reviews undertaken for accepted manuscripts. Reviewers are asked to complete a checklist review form and are provided with free text boxes to elaborate on their assessment. These free text comments are reproduced below.

ARTICLE DETAILS

Title (Provisional)

Prevalence of presbyopia among social safety net beneficiaries with the cognitive, numeracy, and dexterity skills required for smartphone use: a cross-sectional analysis of THRIFT RCT screening data from Kurigram, Bangladesh

Authors

Aftab , Ishrat Binte; Chakma, Tisha; Pant, Sonia; Sigwadhi, Lovemore Nyasha; Shitol, Sharmin Akter; Rahman, H. M. Masudur; Alam, Jahangir; Haque , Enam; Chadalavada, Harithaa P; Murtaza, Fahmeeda; Chan, Ving Fai; Little, Julie-Anne; Khanna, Rohit C; MacKenzie, Greame; Gudwin, Ella; Lohfeld, Lynne; Clarke, Mike; Shonchoy, Abu; Congdon, Nathan; Rabbani, Atonu

---

VERSION 1 - REVIEW

**Reviewer**	**1**
**Name**	**Kelson, Zoe**
**Affiliation**	**University of Exeter, Mathematics**
**Date**	**14-Oct-2025**
**COI**	**None**

This study aims to determine the prevalence of presbyopia among old age and widow allowance recipients who are capable of using smartphones.

Reviewer comments:

"This study analyzed data from the THRIFT RCT in two subdistricts of Kurigram, Bangladesh, using structured screening to identify eligible participants... Of 13,944 beneficiaries screened, 953 participants met eligibility criteria and completed near vision assessments." [Abstract]

and

"This study draws data from the THRIFT RCT 14 screening and recruitment phases in Kurigram Sadar and Nageshwari. These two sub-districts are chosen as OAA and WA allowances are distributed via study partner bKash and vision programs by BRAC and VisionSpring are active there. "

The authors suitably discuss representativeness of the cohort in the study limitations.

Can the authors please confirm if there was any missing data?

"Chi-square and nonparametric trend tests were used to assess group differences and presbyopia prevalencen trend. Robust Poisson regression identified factors independently associated with presbyopia" [Abstract]

and

"Categorical variables were analysed using frequencies and percentages. Chi-squared and Fisher exact test were used to assess the association between presbyopia and categorical variables. Nonparametric trend tests were conducted for ordered variables, while chi-square tests were used for non-ordered categorical variables...

robust Poisson regression was used to assess the significance of associations between presbyopia and potential predictors. Factors with $p < 0.15$ in unadjusted univariable regression were included in a multivariable model. However, mobile type was considered an apriori variable based on this study importance and was included in the multivariable analysis regardless of its p-value (0.271). Adjusted prevalence ratios with 95% CIs were used to measure association."

Appropriate modelling methods have been applied.

Can the authors please confirm if and how normality was assessed prior to the choice of tests applied?

Can the 'nonparametric trend test' please be specified here by the authors?

"Table 1: Descriptive Statistics of the Study Participants"

Can race and ethnicity please be reported in this table?

Can data on comorbidities please be considered for inclusion in the study?

"A limitation is that participants represented the lower socio-economic strata of society, therefore, do not provide population-representative sample. Nonetheless, they constitute a group with a unique need to access mobile financial services. These results are representative only of this cohort and geographic region of Bangladesh, and our results cannot be applied to other settings with confidence."

A discussion on the study limitations is provided by the authors.

Thanks for providing a copy of the STROBE checklist.

**Reviewer**	**2**
**Name**	**Turkoglu, Ceren**
**Affiliation**	**Lokman Hekim University**
**Date**	**22-Nov-2025**

COI None

Reviewer Report: The prevalence of presbyopia among older social safety net beneficiaries in Bangladesh (bmjopen-2025-108327)

Overall Impression and Decision

This is an Original Research article that addresses a highly relevant public health issue: the burden of uncorrected presbyopia among a vulnerable population in Bangladesh who rely on mobile financial services (MFS) for social safety net benefits. The study is well-motivated, drawing on data from a larger Randomized Controlled Trial (RCT) (THRIFT), and the findings—a high prevalence of presbyopia (62.6%) and its association with female gender and Widows' Allowance status—are important for informing policy on digital financial inclusion.

However, the manuscript, in its current form, suffers from several major methodological and reporting limitations that must be addressed before it can be considered for publication. The most critical issue is the highly selective and non-representative nature of the study sample, which severely limits the generalizability of the prevalence estimate.

Decision: Major Revision Required

The authors must thoroughly address the major comments below, particularly those concerning the sampling frame, selection bias, and the interpretation of the prevalence estimate.

Major Comments

1. Sampling and Selection Bias (Critical Flaw):

◦ The reported prevalence of 62.6% is not a generalizable estimate for the target population. The study explicitly states that participants were selected based on a highly restrictive, multi-step screening process for the THRIFT RCT, which included criteria like:

◦ Being an OAA/WA recipient in specific sub-districts.

◦ Residing in a bKash catchment area.

◦ Being in the lowest three wealth quintiles.

◦ Exclusion of those already independently using bKash mobile banking.

◦ Passing a Mobile Phone Readiness Test (Numeracy, Dexterity, and Cognition - NDC test) with a minimum score of 8/10.

◦ Recommendation: The authors must fundamentally reframe the paper. The current title and abstract imply a general prevalence study, which is misleading. The prevalence is only representative of a highly selected subgroup of OAA/WA beneficiaries who are poor, not currently using MFS, and cognitively/functionally capable of using a smartphone. This must be made clear in the title, abstract, introduction, and discussion. The authors should use

terms like “prevalence among a pre-screened, RCT-eligible cohort” or “prevalence among a subset of beneficiaries capable of using smartphones.”

2. Denominator and Generalizability:

◦ The study screened 13,944 beneficiaries, but only 953 participants met the eligibility criteria and completed the near vision assessments. This represents a massive exclusion rate (over 93%).

◦ Recommendation: The Methods section must include a detailed flowchart (Figure 1) showing the number of exclusions at each step of the screening process (e.g., how many failed the wealth screen, how many were excluded for already using bKash, how many failed the NDC test). This is crucial for transparency and for readers to understand the study’s internal validity and external generalizability. The current text mentions Figure 1, but the figure itself is not present in the provided pages, and the description in the text is insufficient.

3. Interpretation of Age-Related Findings:

◦ The result states that the highest prevalence (62.6%) was seen in the 45–54 age group and that it “declined with an increasing age” (Key Messages). This finding is counter-intuitive for an age-related condition like presbyopia, which typically increases in prevalence and severity with age.

◦ Recommendation: The authors must provide a clear, evidence-based explanation for this decline in the Discussion. Is it due to: a) Survival bias (less healthy/sighted individuals in older groups were excluded)? b) Selection bias (older individuals were more likely to fail the NDC test and thus were excluded)? c) Spectacle wear bias (older individuals may have already acquired corrective lenses, thus lowering the uncorrected prevalence)? This point needs significant discussion.

4. Statistical Analysis and Reporting:

◦ The authors used Robust Poisson regression to estimate Adjusted Prevalence Ratios (APR), which is appropriate for common outcomes like presbyopia. However, the reporting is sparse.

◦ Recommendation: The manuscript must include a full table of the multivariable regression results (APR, 95% CI, and p-value) for all variables included in the final model, not just the significant ones (female gender and WA status). This is standard practice for reporting regression models.

Minor Comments

1. Clarity of Definition:

◦ The definition of presbyopia is given as: “binocular presenting near visual acuity of N6.3 or worse, correctable to at least N5 with near vision glasses, and with near vision of >= 6/12 in both eyes.”

◦ Recommendation: The phrase “and with near vision of >= 6/12 in both eyes” seems to be a criterion for eligibility or correctability, not the definition of presbyopia itself. Please clarify this definition. Does it mean the distance vision must be �? If so, state it clearly as distance vision.

2. Terminology Consistency:

◦ The abstract and key messages use “Widows’ Allowance (WA) recipients,” but the results and discussion should consistently use the full name or the abbreviation.

◦ Recommendation: Ensure consistent use of “OAA” and “WA” throughout the text.

3. Introduction/Background:

◦ The Introduction is strong in linking presbyopia to MFS use. However, the transition to the study objective is slightly abrupt.

◦ Recommendation: Briefly mention the specific gap this study fills—namely, the prevalence among the specific subgroup of beneficiaries who are candidates for an MFS intervention.

4. Results Presentation:

◦ The results section mentions a Table 1 (Descriptive Statistics) and a Figure 2 (Presbyopia prevalence by age and gender), but these are not provided in the pages I have reviewed.

◦ Recommendation: Ensure all referenced tables and figures are included and clearly labeled. The text must stand alone, but the figures are essential for supporting the claims, especially the counter-intuitive age trend.

Summary of Required Revisions

The authors must:

1. Refocus the manuscript to clearly state that the prevalence is for a highly selected, RCT-eligible subgroup (Major Comment 1).

2. Provide a detailed participant flow chart (Figure 1) showing the number of exclusions at every step of the screening process (Major Comment 2).

3. Provide a robust explanation for the decline in presbyopia prevalence in the oldest age groups (Major Comment 3).

4. Include a full table of the multivariable regression results (Major Comment 4).

5. Clarify the vision screening criteria (Minor Comment 1).

If these major revisions are satisfactorily addressed, the manuscript will be a valuable contribution to the literature on vision and digital financial inclusion.

VERSION 1 - AUTHOR RESPONSE

REVIEWER 1 COMMENTS (Prof. Zoe Kelson)

1. "This study analyzed data from the THRIFT RCT in two subdistricts of Kurigram, Bangladesh, using structured screening to identify eligible participants... Of 13,944 beneficiaries screened, 953 participants met eligibility criteria and completed near vision assessments." [Abstract]
and
"This study draws data from the THRIFT RCT 14 screening and recruitment phases in Kurigram Sadar and Nageshwari. These two sub-districts are chosen as OAA and WA allowances are distributed via study partner bKash and vision programs by BRAC and VisionSpring are active there. "
The authors suitably discuss representativeness of the cohort in the study limitations.

Can the authors please confirm if there was any missing data?

Response: We thank the reviewer for the need of the clarification on missing data. We have added a footnote on our Table 1 that data for all the variables were available for all participants.

2. "Chi-square and nonparametric trend tests were used to assess group differences and presbyopia prevalence trend. Robust Poisson regression identified factors independently associated with presbyopia" [Abstract]
and
"Categorical variables were analysed using frequencies and percentages. Chi-squared and Fisher exact test were used to assess the association between presbyopia and categorical variables. Nonparametric trend tests were conducted for ordered variables, while chi-square tests were used for non-ordered categorical variables...
robust Poisson regression was used to assess the significance of associations between presbyopia and potential predictors. Factors with $p < 0.15$ in unadjusted univariable regression were included in a multivariable model. However, mobile type was considered an apriori variable based on this study importance and was included in the multivariable analysis regardless of its p-value (0.271). Adjusted prevalence ratios with 95% CIs were used to measure association."
Appropriate modelling methods have been applied.
Can the authors please confirm if and how normality was assessed prior to the choice of tests applied?
Can the 'nonparametric trend test' please be specified here by the authors?

Response: We thank the reviewer for the request of further clarification on normality assessment. Only age was assessed for normality since it was the only continuous variable in

the analysis. The Shapiro Wilk test was used for normality assessment and we have added this information to our description of the main analysis. We have added that Linear by Linear trend tests were done to assess trend-ordered variables (age categories, educational level, and national quintile), while Chi-Squared tests were done to assess trend for non-ordinal variables. We have made the necessary changes in the analysis section in the manuscript as follows:

"Categorical variables were analysed using frequencies and percentages. The Shapiro-Wilk test was used for normality assessment. Chi-squared and Fisher exact test were used to assess the association between presbyopia and categorical variables. Linear by linear trend tests were conducted for ordered variables to assess trend across age categories, educational level and national quintiles, while chi-square tests were used for non-ordered categorical variables to assess trend across the categories."

3. "Table 1: Descriptive Statistics of the Study Participants"
Can race and ethnicity please be reported in this table?
Can data on comorbidities please be considered for inclusion in the study?

Response: We appreciate the reviewer's suggestion to enrich Table 1 with additional participant characteristics.

Race and Ethnicity

The study population consists of OAA and WA beneficiaries of Bangladesh government's social safety net program. Essentially the entire population belongs to a single national ethnic group, and official census for this cohort does not capture data on race and ethnicity. Thus we did not collect race/ethnicity data, as it would not provide meaningful information and variability within the sample. To clarify this, we have added this statement in the Methods section ***"Race/ethnicity data were not collected as effectively the entire cohort belongs to a single national ethnic group."***

Comorbidities

Including comorbidities are common in many geriatric studies, however, this trial was specifically designed to evaluate the impact of presbyopia correction and digital financial training on Mobile Financial System use. The only health assessments we focused on were relevant to vision-related status, dexterity, numeracy, and cognitive ability necessary for smartphone operation. Given the limited relevance of comorbidities to our primary outcome, we haven't added this in table 1. To address this, we will add this sentence in the limitation part of the discussion, ***"Moreover, Given the limited relevance of systemic comorbidities to the primary study outcome, we have not chosen to include data on comorbidities in our analyses. Future investigations can include comprehensive health assessments to explore potential effect modification."***

REVIEWER 2 COMMENTS (Dr. Ceren Turkoglu)

Major Comment 1 — Sampling and Selection Bias (Critical Flaw)
"◦ The reported prevalence of 62.6% is not a generalizable estimate for the target population. The study explicitly states that participants were selected based on a highly restrictive, multi-step screening process for the THRIFT RCT, which included criteria like:
◦ Being an OAA/WA recipient in specific sub-districts.
◦ Residing in a bKash catchment area.
◦ Being in the lowest three wealth quintiles.
◦ Exclusion of those already independently using bKash mobile banking.
◦ Passing a Mobile Phone Readiness Test (Numeracy, Dexterity, and Cognition - NDC test) with a minimum score of 8/10.
◦ Recommendation: The authors must fundamentally reframe the paper. The current title and abstract imply a general prevalence study, which is misleading. The prevalence is only representative of a highly selected subgroup of OAA/WA beneficiaries who are poor, not currently using MFS, and cognitively/functionally capable of using a smartphone. This must be made clear in the title, abstract, introduction, and discussion. The authors should use terms like "prevalence among a pre-screened, RCT-eligible cohort" or "prevalence among a subset of beneficiaries capable of using smartphones."

Response: Thanks to the reviewers for the recommendation. We have revised the title and abstract.

Regarding the comment about population representativeness, it was never the goal of this research to carry out a population-based assessment of presbyopia prevalence. We have now made it clearer in our Abstract, Introduction, Methods and Discussion that the goal of this research is to assess the prevalence of presbyopia is a highly-relevant and economically-important vulnerable cohort: recipients of Bangladeshi government safety net payments, who must access these payments through online banking, as through a smart phone. Presbyopia is a particularly meaningful disability in this very large population.

Our strengths and weaknesses section after the Abstract now features the following additional language to further underscore this point:

The study sample reflects an economically important and vulnerable group receiving government benefits, and is not meant to be population-representative.

Major Comment 2 — Denominator and Generalizability:
"The study screened 13,944 beneficiaries, but only 953 participants met the eligibility criteria and completed the near vision assessments. This represents a massive exclusion rate (over 93%).
◦ Recommendation: The Methods section must include a detailed flowchart (Figure 1)

showing the number of exclusions at each step of the screening process (e.g., how many failed the wealth screen, how many were excluded for already using bKash, how many failed the NDC test). This is crucial for transparency and for readers to understand the study's internal validity and external generalizability. The current text mentions Figure 1, but the figure itself is not present in the provided pages, and the description in the text is insufficient."

Response: We thank the reviewers for the recommendation. We already uploaded the flowchart with the original submission in a separate file named as "Figure 1" that shows the flow of data, number of exclusions at each step of the screening process (social screening, wealth quintile screening, MFS use and NDC test and vision screening) including the reasons. We have now corrected few percentage values shown in parentheses in figure 1. The original counts remain unchanged. The revised figure has been updated accordingly. The flowchart is reproduced here for reference:

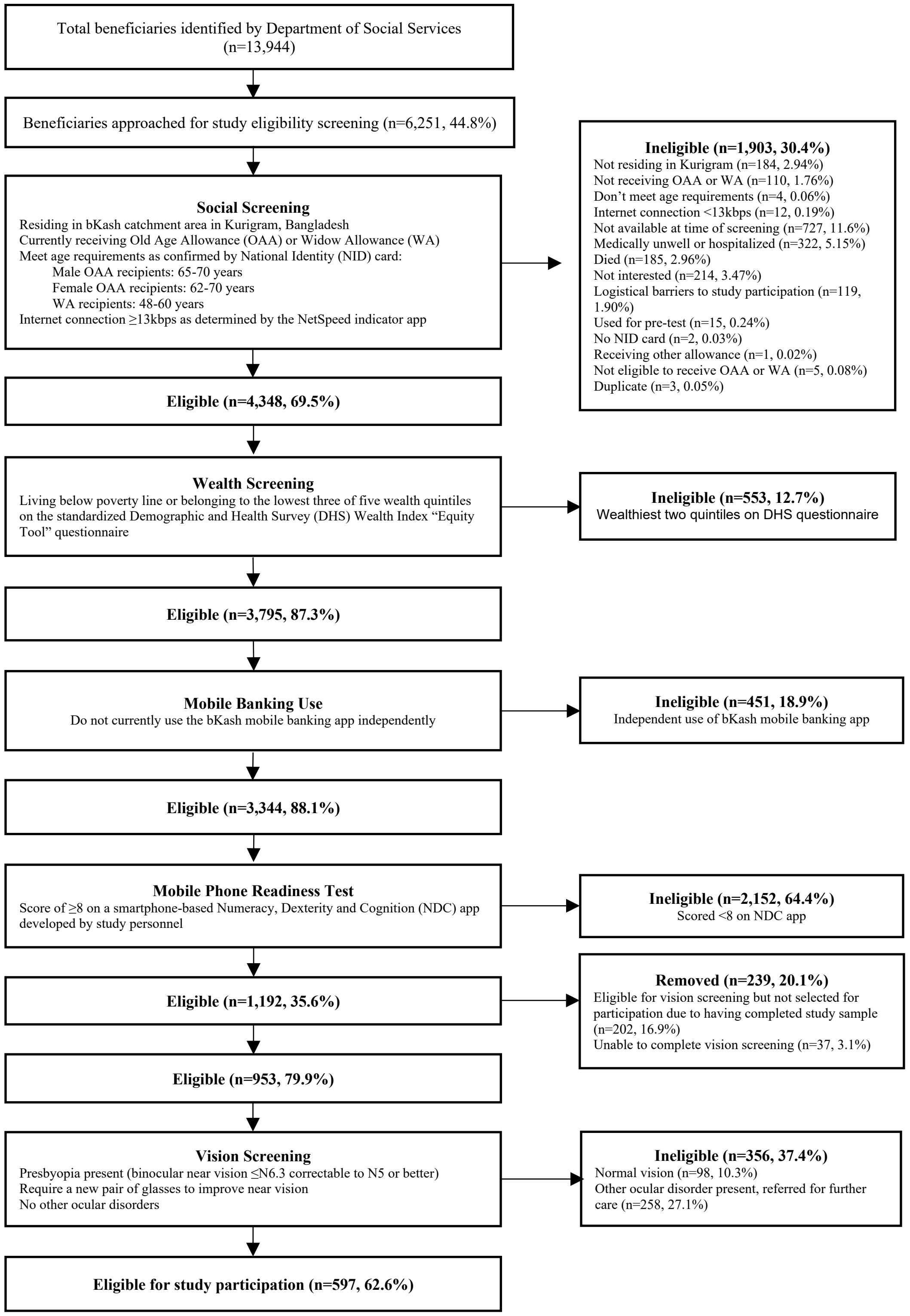

Figure 1: Progress of potential participants through the study

Major Comment 3 — Interpretation of Age-Related Findings

“The result states that the highest prevalence (62.6%) was seen in the 45–54 age group and that it “declined with an increasing age” (Key Messages). This finding is counter-intuitive for an age-related condition like presbyopia, which typically increases in prevalence and severity with age.
◦ Recommendation: The authors must provide a clear, evidence-based explanation for this decline in the Discussion. Is it due to: a) Survival bias (less healthy/sighted individuals in older groups were excluded)? b) Selection bias (older individuals were more likely to fail the NDC test and thus were excluded)? c) Spectacle wear bias (older individuals may have already acquired corrective lenses, thus lowering the uncorrected prevalence)? This point needs significant discussion.”

Response: In fact, the decline of presbyopia with age is widely-reported in large studies, see for example

He M, Abdou A, Ellwein LB, Naidoo KS, Sapokta YD, Thulasiraj RD, Varma R, Zhao J, Kocur I, Congdon N. Age-related prevalence and met need for correctable and uncorrectable near vision impairment in a multi-country study. Ophthalmology. 2014;121:417-22.

The authors agree that it appears counter-intuitive initially, but the reason is fairly straightforward: The definition of presbyopia requires that impaired near vision occur in the context of normal distance vision, otherwise it cannot be distinguished from conditions such as cataract, affecting both near and distance vision, that are also prevalent in older populations. As the prevalence of conditions such as cataract rises, that of pure presbyopia actually falls in older populations.

There is no “bias” involved in this widely-reported phenomenon. We have now added the following material to the Discussion:

“The decline in presbyopia per se among older individuals is widely-reported, and is inherent in the definition, which requires that persons with impaired near vision should have normal distance vision, to distinguish between persons with conditions such as cataract, also prevalent in older populations, which affect both near and distance vision. Thus, as the burden of cataract rises in older cohorts, the prevalence of pure presbyopia declines as a direct consequence.”

Major Comment 4 — Statistical Analysis and Reporting:
“The authors used Robust Poisson regression to estimate Adjusted Prevalence Ratios (APR), which is appropriate for common outcomes like presbyopia. However, the reporting is sparse.
◦ Recommendation: The manuscript must include a full table of the multivariable regression

results (APR, 95% CI, and p-value) for all variables included in the final model, not just the significant ones (female gender and WA status). This is standard practice for reporting regression models."

Response: We thank the reviewer for the request. In table 3, we have already added the information for both significant and non-significant variables in the manuscript. We are adding the table 3 here for reference:

Table 3: Factors Associated with Presbyopia, Univariate and Multivariable Analysis

	**Univariate**		**Multivariable**	
Variable	**PR (95 % CI)**	**p-value**	**APR (95% CI)**	**p-value**
**Age (years)**				
45-54	Ref			
55-64	0.91 (0.81-1.03)	0.141		
65+	0.73 (0.66-0.81)	<0.001		
**Sex**				
Male	Ref			
Female	1.35 (1.20-1.51)	<0.001	1.19 (1.02-1.41)	0.031
**Highest level of education**				
Never been to school	Ref			
Completed Primary School (grade 5 pass)	0.92 (0.80-1.06)	0.248		
Completed Secondary/Higher School Certificate	0.80 (0.59-1.09)	0.162		
**Occupation**				
Unemployed	Ref			
Employed	0.87 (0.70-1.07)	0.181		
Retired	0.77 (0.61-0.98)	0.031		
**Mobile type**				
Feature or button	Ref			
Smartphone	1.11 (0.92-1.34)	0.271	1.10 (0.91-1.33)	0.314
**Eyeglasses use**				
No	Ref			
Yes	1.06 (0.94-1.18)	0.343		
**Wealth quintile**				
Quintile 1	1.00 (0.87-1.15)	0.990	0.96 (0.83-1.11)	0.608
Quintile 2	1.11 (0.96-1.29)	0.149	1.11 (0.95-1.29)	0.189
Quintile 3	Ref		Ref	
**Beneficiary type**				
OAA	Ref			
WA	1.33 (1.21-1.47)	<0.001	1.20 (1.04-1.38)	0.010

Notes: Prevalence Ratios (PR) and Adjusted Prevalence Ratios (APR) are presented with their 95% Confidence Intervals (CIs) in parentheses.

Minor Comment 1 — Clarify Definition
"The definition of presbyopia is given as: "binocular presenting near visual acuity of N6.3 or worse, correctable to at least N5 with near vision glasses, and with near vision of >= 6/12 in both eyes."
◦ Recommendation: The phrase "and with near vision of >= 6/12 in both eyes" seems to be a criterion for eligibility or correctability, not the definition of presbyopia itself. Please clarify this definition. Does it mean the distance vision must be >= ? If so, state it clearly as distance vision."

Response: This is our error, the correct definition, as referenced in the above response, should read: ***"binocular presenting near visual acuity of N6.3 or worse, correctable to at least N5 with near vision glasses, and with distance vision of >= 6/12 in both eyes".*** We have made change for the definition in the abstract and in the vision screening section on page 7.

Minor Comment 2 — Terminology Consistency
"◦ The abstract and key messages use "Widows' Allowance (WA) recipients," but the results and discussion should consistently use the full name or the abbreviation.
◦ Recommendation: Ensure consistent use of "OAA" and "WA" throughout the text."

Response: This has been corrected, thank you.

Minor Comment 3 — Introduction/Background
"The Introduction is strong in linking presbyopia to MFS use. However, the transition to the study objective is slightly abrupt.
◦ Recommendation: Briefly mention the specific gap this study fills—namely, the prevalence among the specific subgroup of beneficiaries who are candidates for an MFS intervention."

Response: We thank the reviewer for flagging this issue. We are adding this line before the objective to make the transition smooth. *"Although extensive literature is available on the global burden of uncorrected presbyopia[2], there is a need for specific data on the burden among particular vulnerable populations with an inherent strong need for near vision, such as older persons with a requirement to use smartphones for online banking. The current study is designed to fil this evidence gap by focusing on OAA and WA recipients who are required to access payments through online banking."*

Minor Comment 4 — Results Presentation
"◦ The results section mentions a Table 1 (Descriptive Statistics) and a Figure 2 (Presbyopia prevalence by age and gender), but these are not provided in the pages I have reviewed.
◦ Recommendation: Ensure all referenced tables and figures are included and clearly labeled.

The text must stand alone, but the figures are essential for supporting the claims, especially the counter-intuitive age trend."

Response: We thank the reviewer for the comment. The description statistics table is provided in the manuscript and there is no figure 2, there is table 2 that shows the Prevalence of Presbyopia by Demographic and Socioeconomic Characteristics of Study Participants. The only figure for this manuscript is the flowchart added above in this document that shows the flow of participants through the study. It was added in a separate file as per BMJ Open's policy. Here are Table 1 and Table 2 below for reference, as they appear in the original MS as submitted.

Table 1: Descriptive Statistics of the Study Participants

Characteristic	Total (n=953)	Presbyopic (597)	Non-presbyopic (n=356)	p-value
**Age (years)**				<0.001
45-54	245 (25.7%)	183 (30.7%)	62 (17.4%)	
55-64	202 (21.2%)	138 (23.1%)	64 (18.0%)	
65+	506 (53.1%)	276 (46.2%)	230 (64.6%)	
**Sex**				<0.001
Male	356 (37.4)	183 (30.7)	173 (48.6)	
Female	597 (62.6)	414 (69.3)	183 (51.4)	
**Highest level of education**				0.11
Never attended school	761 (79.9)	487 (81.6)	274 (77.0)	
Completed Primary School	153 (16.1)	90 (15.1)	63 (17.7)	
Completed Secondary School	34 (3.6)	19 (3.2)	15 (4.2)	
Completed Higher Secondary School	5 (0.5)	1 (0.2)	4 (1.1)	
**Literacy level**				0.022
Unable to read or write	684 (71.8)	447 (74.9)	237 (66.6)	
Can read	30 (3.1)	16 (2.7)	14 (3.9)	
Can read and write	239 (25.1)	134 (22.4)	105 (29.5)	
**Occupation**				0.081
Unemployed	34 (3.6)	25 (4.2)	9 (2.5)	
Employed	720 (75.6)	459 (76.9)	261 (73.3)	
Retired	199 (20.9)	113 (18.9)	86 (24.2)	
**Currently have access to a mobile phone**				0.91
Yes	927 (97.3)	581 (97.3)	346 (97.2)	
No	26 (2.7)	16 (2.7)	10 (2.8)	
**Who owns the phone?**				0.25
My own	602 (64.9)	384 (66.1)	218 (63.0)	
Family member	300 (32.4)	185 (31.8)	115 (33.2)	

Neighbor	25 (2.7)	12 (2.1)	13 (3.8)	
**Mobile phone type**				0.31
Smartphone	52 (5.6)	36 (6.2)	16 (4.6)	
Other	875 (94.4)	545 (93.8)	330 (95.4)	
**Internet connection on the phone**				0.63
Yes	70 (7.6)	42 (7.2)	28 (8.1)	
No	857 (92.4)	539 (92.8)	318 (91.9)	
**Eyeglasses use**				0.36
Yes	219 (23.0)	143 (24.0)	76 (21.3)	
No	734 (77.0)	454 (76.0)	280 (78.7)	
**Wealth quintile**				0.13
Quintile 1	516 (54.1)	313 (52.4)	203 (57.0)	
Quintile 2	269 (28.2)	182 (30.5)	87 (24.4)	
Quintile 3	168 (17.6)	102 (17.1)	66 (18.5)	
**Beneficiary type**				<0.001
OAA	515 (54.0)	280 (46.9)	235 (66.0)	
WA	438 (46.0)	317 (53.1)	121 (34.0)	

Notes: Variables were expressed as frequencies with percentages in parentheses.
Data for all variables were available for all participants.

Table 2: Prevalence of Presbyopia by Demographic and Socioeconomic Characteristics of Study Participants

Characteristic	Number	Prevalence (95% CI)	Trend test for association
**Age (years)**			< 0.001
45-54	183	74.7 (68.8-80.0)	
55-64	138	68.3 (61.4-74.7)	
65+	276	54.5 (50.1-58.9)	
**Sex***			< 0.001
Male	183	51.4 (46.1-56.7)	
Female	414	69.3 (65.5-73.0)	
**Highest level of education**			< 0.056
Never been to school	487	64.0 (60.5-67.4)	
Completed Primary School (grade 5 pass)	90	58.8 (50.6-66.7)	
Completed Secondary or Higher School Certificate	20	51.3 (34.8-67.6)	
**Wealth quintile**			0.540
Quintile 1	313	60.7 (56.3-64.9)	
Quintile 2	182	67.7 (61.7-73.2)	
Quintile 3	102	60.7 (52.9-68.1)	
**Beneficiary type***			< 0.001
OAA	280	54.4 (50.0-58.7)	
WA	317	72.4 (67.9-76.5)	

Total	597	62.6 (59.5-65.7)

NB: Non-parametric tests for trend for ordered categorical variables

* Chi-square test was used to assess the group comparison for non-ordered categorical variables.

VERSION 2 - REVIEW

**Reviewer**	**2**
**Name**	**Turkoglu, Ceren**
**Affiliation**	**Lokman Hekim University**
**Date**	**30-Jan-2026**
**COI**	

Thank you for giving me the opportunity to review the revised version of the manuscript.

I acknowledge that the authors have made revisions in response to the previous comments, and I appreciate their efforts. Based on the current version, I am willing to recommend acceptance of the manuscript.

However, I would like to note that some of the revisions do not fully address the concerns raised in the initial review, and therefore the changes are not entirely satisfactory in all aspects. While these issues may not preclude publication, they should be acknowledged as remaining limitations of the manuscript.

Thank you for considering my evaluation.
